# Inflammation and Dysmetabolism in Systemic Autoimmune Diseases

**DOI:** 10.1155/2019/5438287

**Published:** 2019-07-22

**Authors:** Antonella Afeltra, Antonio Abbate, Gabriele Valentini, Roberto Giacomelli

**Affiliations:** ^1^Unit of Allergology, Immunology and Rheumatology, Department of Medicine, Università Campus Bio-Medico di Roma, Rome, Italy; ^2^Virginia Commonwealth University, USA; ^3^Division of Rheumatology, Department of Precision Medicine, University of Campania “Luigi Vanvitelli”, Naples, Italy; ^4^Division of Rheumatology, Department of Biotechnological and Applied Clinical Science, University of L'Aquila, L'Aquila, Italy

Despite advances in pathogenic and clinical knowledge, rheumatic diseases are still burdened by high morbidity, accrual of irreversible organ damage with development of disability, and increased mortality [1–4]. In the few last decades, interest in the metabolic aspects of rheumatic diseases has gradually increased. The impact of dysmetabolism on rheumatic diseases is complex and extends from pathogenesis to clinical manifestations and potential therapeutic targets.

Metabolic syndrome (MeS) is a cluster of metabolic disorders that includes visceral adipose tissue accumulation, insulin-resistance, alteration in blood cholesterol components and apolipoproteins, and systemic inflammation [5, 6]. The incidence and prevalence of metabolic syndrome is increased in several systemic autoimmune diseases with possible impact on cardiovascular complication and damage accrual [7–9]. One of the possible links between metabolism, MeS, and inflammation is adipokines, a group of cytokines mainly produced by adipose tissue. Consistent literature data clearly demonstrated the involvement of adipokines in autoimmunity and several systemic autoimmune diseases. In this issue, P. Ruscitti et al. deeply reviewed the role of adipokines in the atherogenesis and MeS development in patients with rheumatoid arthritis.

Interleukin-6 (IL-6) is the prototype of a molecular link between inflammation, autoimmunity, metabolism, and adipose tissue [5]. In this issue, A. Laudisio et al. analyzed the impact of olfactory dysfunction on frailty and mortality of elderly patients and demonstrated that this relation could be mediated by IL-6.

Particularly interesting are the implications of Western diet in rheumatic diseases. Polyunsaturated fatty acids (PUFAs) are members of the family of fatty acids, with a wide spectrum of immunological functions: n-6 PUFAs have predominantly proinflammatory features, while n-3 PUFAs seem to exert anti-inflammatory and proresolving properties. We recently reviewed the literature on PUFA in rheumatoid arthritis, showing that n-3-PUFA supplementation could represent an interesting therapeutic option [10]. D-Series resolvins are a product of the metabolism of n-3 PUFA. Crescent data demonstrated the involvement of D-series resolvins and, in particular, resolvin-D1 in immune homeostasis. In general, resolvin-D1 seems to downregulate the production of proinflammatory cytokines from T helper 1 and T helper 17 lymphocytes and to promote the differentiation of T regulatory cells. However, only few data are available on the role of resolvins in systemic autoimmune diseases. In a paper published in this special issue, L. Navarini et al. demonstrated a marked reduction of resolvin-D1 levels in patients affected by Systemic Lupus Erythematosus (SLE) compared to the general population, especially in association with low complement levels. These findings suggest a specific role of bioactive lipids in SLE [11].

Another relevant topic in the field of relation between inflammation and metabolism is represented by bile acids. Bile acids play a pivotal role in intestinal absorption of fatty acids and in delivery of fatty acids to the apical membrane of enterocytes. K. Uchiyama et al. presented in this special issue a review of the available evidences on the implication of dietary lipids and fatty acids malabsorption in Crohn disease.

Overall, crescent data demonstrated the involvement of metabolism in several aspects of systemic autoimmune diseases with interesting implication for disease prevention, optimization of disease management, and drug development.
